# Production of functional CD19 CAR T cells under hypoxic manufacturing conditions

**DOI:** 10.3389/fimmu.2025.1675786

**Published:** 2025-10-08

**Authors:** Isabella Micallef Nilsson, Thomas Poiret, Jinhye Ryu, Mohammadali Mohammadpour, Johan Henriksson, Anders Österborg, Jonas Mattsson, Anna Schurich, Isabelle Magalhaes

**Affiliations:** ^1^ Department of Oncology-Pathology, Karolinska Institutet, Stockholm, Sweden; ^2^ Center for Hematology and Regenerative Medicine, Department of Medicine Huddinge, Karolinska Institutet, Stockholm, Sweden; ^3^ Department of Clinical Science, Intervention and Technology, Karolinska Institutet, Stockholm, Sweden; ^4^ Department of Molecular Biology, Umeå Centre for Microbial Research, Umeå University, Umeå, Sweden; ^5^ Department of Hematology, Lymphoma Unit, Karolinska University Hospital, Stockholm, Sweden; ^6^ Gloria and Seymour Epstein Chair in Cell Therapy and Transplantation, Princess Margaret Cancer Centre and University of Toronto; Princess Margaret Cancer Centre, University Health Network, Toronto, ON, Canada; ^7^ Department of Infectious Diseases, School of Immunology and Microbial Sciences, King’s College London, London, United Kingdom; ^8^ Department of Clinical Immunology and Transfusion Medicine, Karolinska University Hospital, Stockholm, Sweden

**Keywords:** CAR T cells, hypoxia, CLL (chronic lymphocytic leukemia), tumor microenvironment, mitochondria

## Abstract

**Introduction:**

Chronic lymphocytic leukemia (CLL) has proven difficult to treat with chimeric antigen receptor (CAR) T cell therapy. CLL cells can negatively alter T cell fitness and induce a pseudohypoxic state. We hypothesized that production of CAR T cells under restricted oxygen conditions resembling physiological oxygen levels that can be encountered in tissues (*i.e.* 2% O_2_) could promote outgrowth of hypoxia-tolerant CAR T cells.

**Methods:**

We performed *in vitro* phenotypic and functional assessments of CD19-directed CAR T cells produced in either 21% (^Nor^CAR) or 2% (^Hyp^CAR) O_2_ derived from healthy donors (HDs) or patients with CLL.

**Results:**

Production of HD-derived CAR T cells in 2% O_2_ promoted the enrichment of a naïve-like subset. ^Hyp^CAR and ^Nor^CAR cells were functionally distinct; CD4+ ^Hyp^CAR cells produced more IL-2 and tumor necrosis factor than CD4+ ^Nor^CAR cells. Production in 2% O_2_ was not detrimental to viability or proliferation upon cognate antigen-stimulation and led to increased activation. After chronic stimulation in hypoxia, ^Hyp^CAR-product remained enriched in naïve-like cells, and demonstrated cytotoxic and cytokine production capacity. In CAR T cells derived from patients with CLL, ^Nor^CAR and ^Hyp^CAR subsets were functionally and phenotypically comparable, but displayed different mitochondrial metabolism.

**Discussion:**

We demonstrated that production in 2% O_2_ is not detrimental, confers subtle but lasting functional and phenotypic changes in CAR T cells warranting further research on the impact of hypoxic production on CAR T cell functionality in hypoxic tumor microenvironments.

## Introduction

Chimeric antigen receptor (CAR) T cell therapy has transformed the prognostic outlook for a variety of relapsed and treatment-refractory hematological malignancies (1). Chronic lymphocytic leukemia (CLL) has been a particular challenge as regards production of clinically effective CAR T cells, as CLL induces immune dysfunction, making it difficult to obtain robust T cell populations for CAR production (2). Recently, lisocabtagene maraleucel (Breyanzi^®^) became the first CAR-product to earn FDA approval for CLL treatment (3). Though significant progress has been made in the management of CLL and other hematological malignancies with CAR T cells, most patients treated with commercially available CAR-products do not experience durable, complete remission (4), one reason for which is thought to be the hypoxic tumor microenvironment (TME) (5).

Hypoxia refers to reduced oxygen availability relative to normal oxygen levels in a given tissue (6), usually |0-1.5% (7, 8). Hypoxia is a hallmark of the TME, most tumors displaying levels <2% O_2_ and malignant transformation can lower oxygen levels in the lymphoid niche (9). Atmospheric oxygen levels (21% O_2_), in contrast, are significantly higher than those encountered *in situ* where O_2_ is an average around 5% in tissues (10). Culture at atmospheric oxygen levels is not only physiologically irrelevant; it can lead to oxidative stress, DNA damage, and increased inflammatory signaling (8), and may skew the cellular phenotype and function and select cells less adapted to reintroduction to lower oxygen levels. Performing *in vitro* assessment of CAR T cells at 21% O_2_ is of limited translational value, and the production of CAR T cells at 21% O_2_ might have a negative impact on CAR-product quality (8, 11). Some studies have reported exposure to hypoxia during CAR T cell production, throughout either the expansion phase (12) or the activation phase (13) but not for the entire production cycle. Here, we hypothesized that producing CD19-directed CAR T cells in 2% O_2,_ for the entire duration of the production cycle would lead to the outgrowth of a subset of cells tolerant to lower oxygen levels or, alternatively, prime cells for reintroduction into a lower-oxygen environment. We examined the effect of reduced oxygen on the production of CAR T cells derived from healthy donors (HDs) and patients with CLL.

## Materials and methods

### CAR T cell production and phenotyping

Peripheral blood mononuclear cells (PBMCs) were isolated from buffy coats (purchased from Karolinska University Hospital, Huddinge, Sweden) of anonymized adults HD blood donors who met standard donation criteria and gave written, informed consent in accordance with Institutional guidelines. CAR T cells were produced as described previously (14) using a γ−retroviral vector encoding for an anti-CD19 CAR containing a CD28 co-stimulatory domain (kindly provided by Prof. S. Rosenberg, NCI, Bethesda, USA). Cells were cultured in CO_2_ incubators at 2% O_2_ (^Hyp^CAR) using a ICO50 CO_2_ incubator (Memmert), where oxygen levels are controlled by introducing nitrogen; or 21% O_2_ (^Nor^CAR) using a Forma Steri-Cycle incubator (Thermo Scientific) in AIM-V medium (Gibco) supplemented with 300 IU/mL IL-2 (Novartis), and 10% fetal bovine serum (FBS, Gibco), or 5% human AB serum (HS, Sigma-Aldrich). Cells were maintained in the respective incubators for the duration of production, except during cell media changes and centrifugation steps (including the transduction step). Patient samples: Blood samples were obtained from patients with CLL (median age 76.5 years, 52–87 years) (Karolinska University Hospital). CAR production was performed as described above. Informed written consent was obtained from all patients and the ethical approval (permit DNR 00-138) obtained from the national ethics authority (https://etikprovningsmyndigheten.se).

CAR T cell production yields a mix of transduced cells expressing CARs and non-transduced cells that do not express CARs. Our results either indicate “CAR T cells”/”CAR+”, referring to cells confirmed by flow cytometry to express CARs, or “CAR-product” (not gated specifically on CAR T cells). Cells were analyzed by flow cytometry (CytoFLEX, Beckman Coulter and FACS Canto, Becton Dickinson) using antibodies and dyes, as indicated in **Supplementary Tables 1–3**.

### Human cancer cell line

K562 chronic myelogenous leukemia cell lines expressing CD19 (K562-CD19+) or NGFR (K562-NGFR+) (kindly provided by Dr. S. Feldman, National Cancer Institute, Bethesda, MD) were kept in RPMI-1640 (Hyclone) supplemented with 10% FBS and 1% penicillin-streptomycin (Hyclone). K562-CD19+ cells were transduced with green fluorescent protein (GFP)/firefly luciferase fusion protein (GFP/luciferase; SFG vector, kindly provided by Prof. M. Sadelain, Memorial Sloan Kettering Cancer Center, New York City, NY, USA) to generate K562-CD19+Luc^+^. Expression of CD19 and GFP was confirmed using flow cytometry (CytoFLEX).

### Cytotoxicity assays

The cytotoxicity of HD-derived CAR-products was evaluated against K562-CD19+Luc^+^. Donor-matched untransduced T cells were used as negative controls. Co-cultures were set up at 1:2. 1:4, and 1:10 effector (CAR+ T cells): target (tumor cells) E:T ratios. Co-cultures were maintained in AIM-V medium supplemented with 5% HS and 300 IU/mL IL-2 for 24 h at 21% or 2% O_2_. Cell suspension was combined with ONE-Glo EX Luciferase Assay System reagent (Promega) according to manufacturer’s instructions, and readout performed using a CLARIOstar Multireader (BMG Labtech). Killing is calculated as follows:


$$\%\;specific\;killing=100\;\times \;(1-\frac{CAR\;T\;cell\;biolum\;product}{Untransduced\;T\;cell\;biolum})$$

Cytotoxicity of CLL-derived CAR T cells was evaluated against K562-CD19+ cells using chromium-51 as described previously (15). Following 4 hours of co-culture at 21% O_2_ (no hypoxic incubator is available for incubation of radioactive products in our facility), 37°C and 5% CO_2_, chromium release was detected on a 1450 MicroBeta Liquid Scintillation counter (Perkin Elmer). Killing is calculated as follows:


$$\begin{array}{c} \%\;specific\;lysis\\ =100\;\times \;\frac{sample\;counts\;per\;minute/spontaneous\;release}{maximum\;release/spontaneous\;release} \end{array}$$

### Cytokine production

CAR T cells were co-cultured at a 1:1 E:T ratio with K562-CD19+ in AIM-V supplemented with 5% (HS) in the presence of brefeldin A (Sigma-Aldrich), GolgiStop (BD Biosciences), and anti-CD107a (BD Biosciences) antibody for 6 h at 37°C and 21% or 2% O_2_. Cell surface staining was performed after incubation, followed by fixation and permeabilization with BD Cytofix/Cytoperm kit (BD Biosciences) and intracellular staining. PMA/ionomycin (Sigma-Aldrich) and K562-NGFR+ cells were used as positive and negative controls, respectively. Cells were analyzed by flow cytometry (CytoFLEX).

### Short-term *in vitro* restimulation assays

CAR T cell transduction efficiency, phenotype, and mitochondrial characteristics were determined using flow cytometry (CytoFLEX) prior to the start of the assay (T0). ^Nor^CAR- and ^Hyp^CAR T cells were stimulated using irradiated (55 Gy, CIX2 X-ray cabinet, Xstrahl) K562-CD19+ cells in a 1:1 effector:target (E:T) ratio according to one of two timelines; in the first timeline, the CAR-product was analyzed 24 h after the first stimulation (T1), restimulated 24 h later, and analyzed 24 h after the second stimulation (T2). Following the second timeline, CAR-product was stimulated, and analyzed by flow cytometry for immune checkpoints expression 72 hours later (T1). At the same time, cytotoxicity was evaluated after restimulation and One-Glo EX Luciferase Assay as described above. Remaining CAR-product was stimulated again and assessed 72 h later for immune checkpoints expression, cytotoxicity, and cytokine production (as described previously) (T2). Remaining CAR-product were stimulated again and interrogated (immune checkpoints, cytotoxicity, and intracellular cytokine assay) 72 hours later (T3). ^Hyp^CAR T cells were stimulated in 2% O_2_ (^Hyp-Hyp^CAR), and ^Nor^CAR T cells were stimulated in 21% O_2_ (^Nor-Nor^CAR) or 2% O_2_ (^Nor-Hyp^CAR) to recapitulate a clinical-like setting wherein CAR T cells are produced in 21% O_2_ and encounter lower oxygen levels after infusion. Donor-matched CAR T cells co-cultured with K562-NGFR+ (nonspecifically stimulated), alone (unstimulated), or donor-matched untransduced T cells were used as negative controls. Co-cultures were maintained in AIM-V medium supplemented with 10% FBS or 5% human AB serum and IL-2 (300 IU/mL).

### Data analysis, visualization, and statistics

Flow cytometry data were analyzed using the FlowJo software (FlowJo). GraphPad Prism (Prism 10) was used to perform data analysis and visualization; in the figures, each point represents one donor. Wilcoxon matched-pair signed-rank tests were used to compare two groups of paired samples. Friedman tests followed by Dunn’s multiple comparison tests were performed to compare ≥ 3 paired samples. Numerical values indicated in parentheses represent median values unless otherwise indicated. Statistical significance was set at p <0.05. BioRender (BioRender.com) was used to generate illustrations.

## Results

### ^Hyp^CAR T cells are enriched in naïve-like T cells

**Figure 1:**
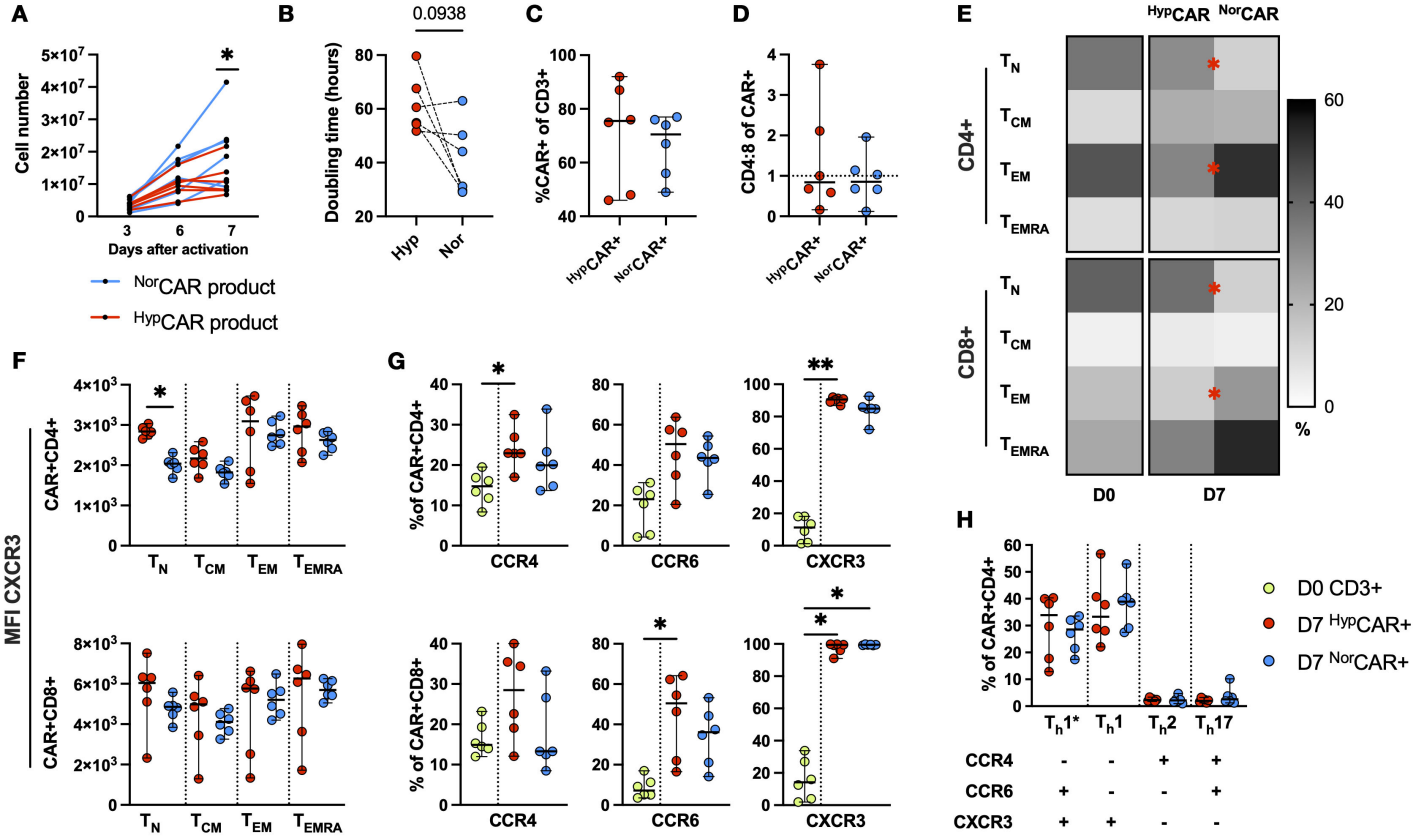
Phenotypic and functional characterization of HD-derived CD19 CAR T cells produced in 2% and 21% O_2_. **(A)** ^Nor^CAR, ^Hyp^CAR-product proliferation at days 3, 6, 7 after transduction. **(B)** Doubling time based on change in cell number between days 3 and 7. **(C)** CAR T cell frequency. **(D)** CD4:8 ratio within ^Nor^CAR and ^Hyp^CAR T cells. **(E)** Memory phenotype of PBMCs on D0 and ^Nor^CAR, ^Hyp^CAR T cells on D7. **(F)** MFI of CXCR3 within memory subsets. **(G)** Frequency of CCR4, CCR6, CXCR3 in PBMCs (D0) and ^Hyp^CAR, ^Nor^CAR T cells (D7). **(H)** T-helper subsets within ^Nor^CAR, ^Hyp^CAR T cells. Median with range are indicated. Wilcoxon tests used for comparisons between two donor-matched conditions; Friedman tests used for three or more. *P < 0.05, **P < 0.01, n=6.

We found similar cell yield between ^Hyp^CAR and ^Nor^CAR-product at days 3 and 6; on day 7, ^Nor^CAR-product contained significantly more cells than ^Hyp^CAR-product (**Figure 1A**). Median doubling times were 37.7 h and 57.7 h (calculated based on change between days 3 and 7), respectively, and while there was no significant difference in doubling times between oxygen conditions, there was a clear tendency for ^Nor^CAR-product to expand more quickly (**Figure 1B**). Production under restricted oxygen levels did not impede transduction efficiency (**Figure 1C**) or significantly favored outgrowth of either CD4+ or CD8+CAR T cells (**Figure 1D**). CD45RA and CCR7 expression was analyzed on the day of activation (D0) and seven days later (D7) (**Figure 1E**). CD4+ T cells present in the PBMCs (start of CAR production) were predominantly naïve-like (T_N_) and effector memory (T_EM_) (T_N_: 36.8%, T_EM_: 44.3%) and CD8+ T cells were primarily T_N_ (41.4%). On D7, CD4+ and CD8+^Hyp^CAR T cells had larger T_N_ populations than ^Nor^CAR T cells (CD4+^Hyp^CAR: 31.3%, CD4+^Nor^CAR: 13.1%; CD8+^Hyp^CAR: 38.3%, CD8+^Nor^CAR: 13.2%) and smaller T_EM_ populations (CD4+^Hyp^CAR, 32.9%; CD4+^Nor^CAR, 52.3%; CD8+^Hyp^CAR, 14%; CD8+^Nor^CAR, 28.3%). T_N_ CD4+^Hyp^CAR T cells expressed higher levels of CXCR3 than the corresponding ^Nor^CAR T cells (**Figure 1F**). When comparing CCR4, CCR6, and CXCR3 expression in D0 T cells and D7 CAR T cells, CXCR3 expression was increased in most (≥72%) CD4+ and CD8+^Hyp^CAR- and ^Nor^CAR T cells (**Figure 1G**), CCR4 expression was increased in CD4+^Hyp^CAR T cells, and CCR6 expression was increased in CD8+^Hyp^CAR T cells compared to that on D0. Based on the expression of these chemokine receptors, we assessed the frequency of the T-helper subsets: Th1* (CCR6^+^CCR4^-^CXCR3^+^) a subset of T cells that produce IFN-γ and low levels of IL-17 (16), Th1 (CCR6^-^CCR4^-^CXCR3^+^), Th2 (CCR6^-^CCR4^+^CXCR3^-^), and Th17 (CCR6^+^CCR4^+^CXCR3^-^) (**Figure 1H**). The majority of cells displayed a Th1 or Th1* phenotype, and there was no difference in the frequency of the respective subsets under different oxygen conditions. No differences were observed in CD25, CD127, CD39, or CD73 expression (**Supplementary Figure 1A**).

### ^Hyp^CAR and ^Nor^CAR-products are functionally distinct

**Figure 2:**
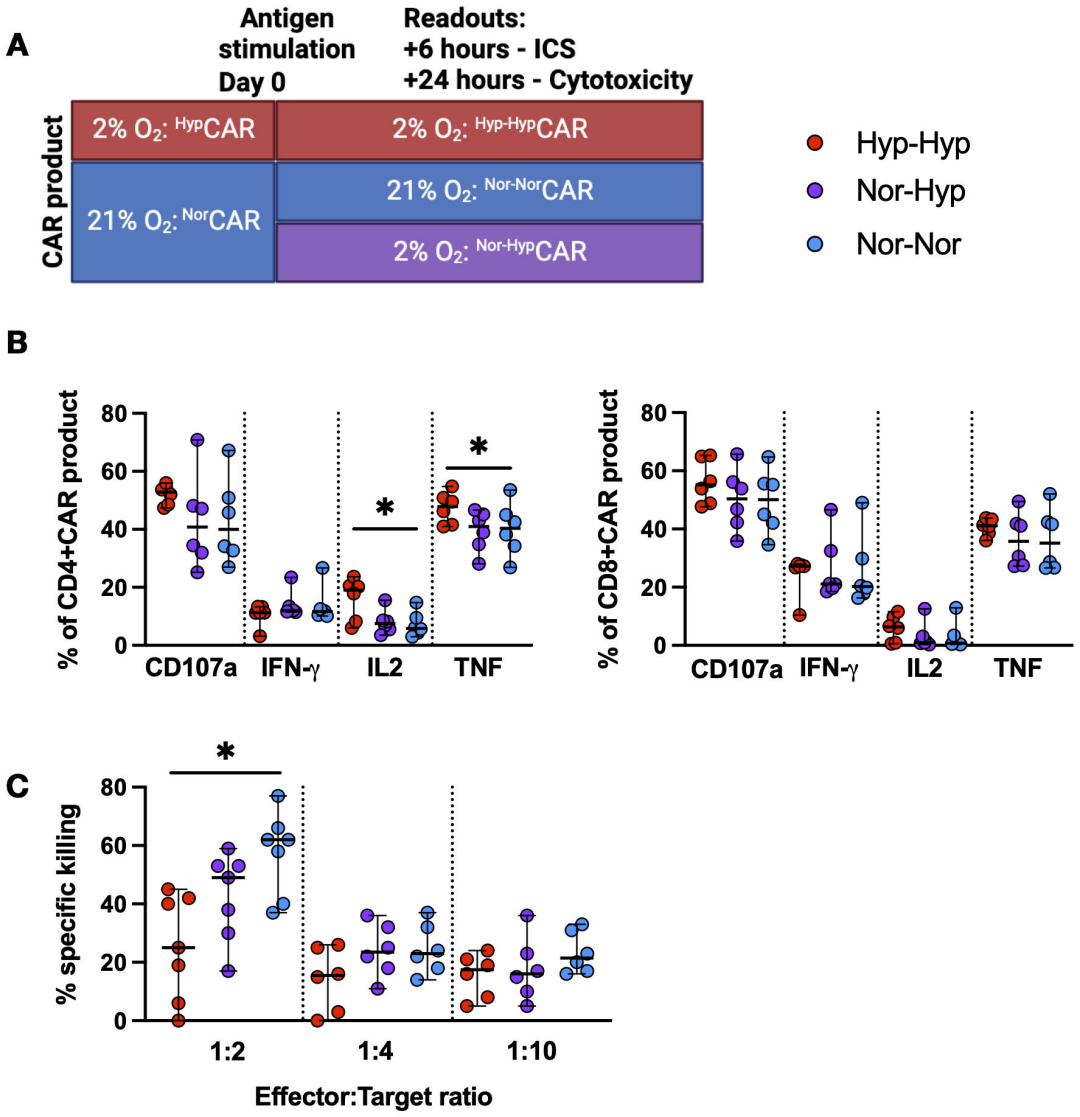
Functional characterization of ^Nor^CAR and ^Hyp^CAR-product. **(A)** Experimental overview of functional assays. **(B)** Cytokine production by CAR-product after co-culture with K562-CD19+. **(C)** Specific killing of K562-CD19+ by CAR-product at 1:2, 1:4, 1:10 effector:target ratios. Median with range are indicated. Friedman tests used for three donor-matched conditions. *P < 0.05, n=6-7.

^Hyp^CAR and ^Nor^CAR-products were stimulated with K562-CD19+ cells in 21% or 2% O_2_ and assessed for cytokine production and cytotoxicity. ^Hyp^CAR-product functionality was evaluated in 2% O_2_ (Hyp-Hyp), and ^Nor^CAR-product functionality was assessed in 21% (Nor-Nor) or 2% O_2_ (Nor-Hyp) (**Figure 2A**). After antigen-specific stimulation, the frequency of degranulating (CD107a expression) and IFN-γ−producing CAR-product was similar between all conditions, but the frequency of IL-2 and TNF production was increased in CD4+^Hyp-Hyp^CAR compared to ^Nor-Nor^CAR-product (**Figure 2B**). No differences were observed between oxygen conditions after PMA/ionomycin stimulation (**Supplementary Figure 2A**). At a 1:2 E:T ratio, ^Nor-Nor^CAR-product was more cytotoxic than ^Hyp-Hyp^CAR-product (**Figure 2C**). There were no differences at 1:4 or 1:10 E:T ratios.

### Production in hypoxia does not impair CAR T cell survival, proliferation, or activation and promotes a naïve-like phenotype after repeated antigen stimulations

**Figure 3:**
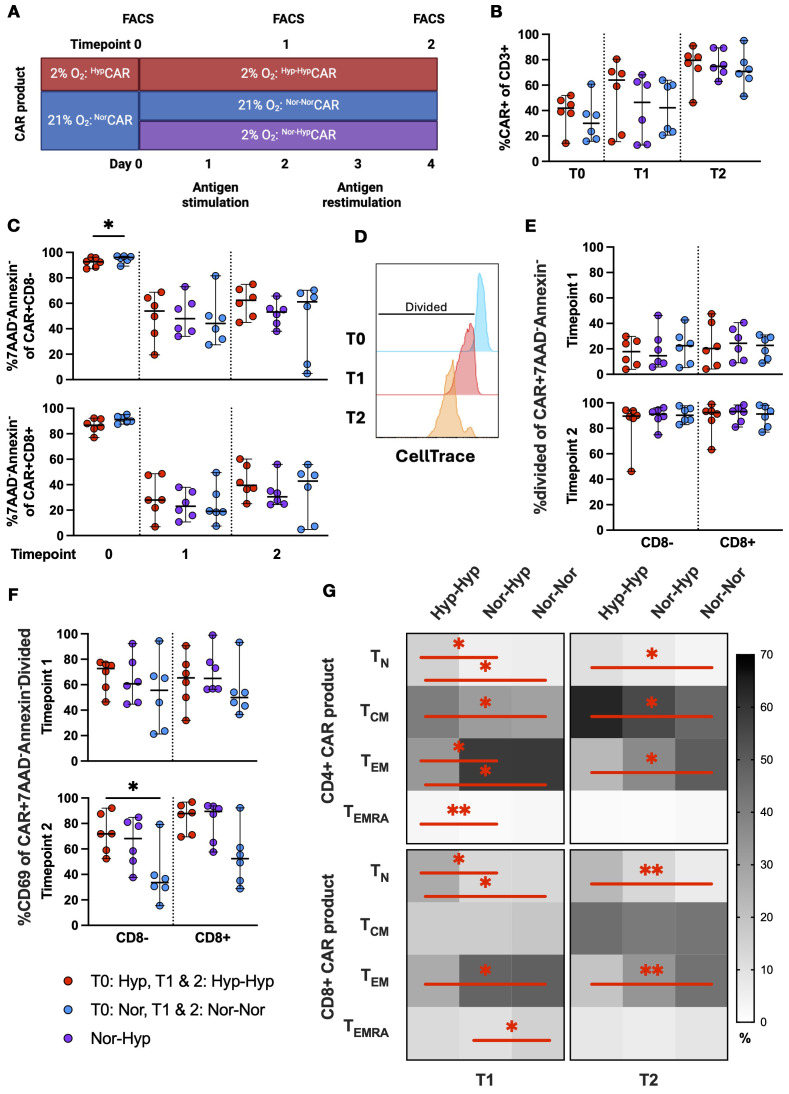
CAR expression, viability, activation, and differentiation phenotype in ^Nor^CAR and ^Hyp^CAR T cells after antigen challenge. **(A)** Experimental overview of short-term *in vitro* restimulation assay. **(B)** CAR expression at T0, T1, T2. **(C)** Frequency of live CAR T cells at T0, T1, T2. **(D)** Representative histogram of CellTrace gating strategy. **(E)** Frequency of divided cells in viable CAR T cells at T1, T2. **(F)** Frequency of CD69+ cells in live, proliferating CAR T cells. **(G)** Differentiation phenotypes of CAR-product at T1 and T2. Median with range are indicated. Wilcoxon tests used for comparisons between two donor-matched conditions; Friedman tests used for three or more. *P < 0.05, **P < 0.01, n=6.

We exposed CAR T cells to repeated stimulations with K562-CD19+ cells in 2% or 21% O_2_ assessing cells by flow cytometry prior to antigen exposure (T0) after one (T1) and two (T2) stimulations (**Figure 3A**). In this analysis, CD8neg was used as a surrogate for the CD4+ population (no anti-CD4 antibody was included in the panel).

At T0, the median CAR expression was 30% among ^Nor^CAR T cells and 41.9% among ^Hyp^CAR T cells (**Figure 3B**). CAR expression increased after each stimulation (T1: ^Nor-Nor^CAR T 42.2%, ^Nor-Hyp^CAR T 46.4%, ^Hyp-Hyp^CAR T 64.1%; T2 ^Nor-Nor^CAR T 70.8%, ^Nor-Hyp^CAR T 74.9%, ^Hyp-Hyp^CAR T 79.6%) and also increased over time in unstimulated cells, albeit less so than among stimulated cells (**Supplementary Figure 3A**). At T0, the frequency of live, non-apoptotic cells (7AAD^-^Annexin^-^) was high in both CD8neg and CD8+^Nor^CAR and ^Hyp^CAR T cell subsets (>86%) (**Figure 3C**). After stimulation, viability decreased under all conditions (particularly for CD8+CAR T cells), with no difference in viability between oxygen conditions in either CD8neg or CD8+CAR T cell subsets. We examined the proliferation of live cells after stimulation by measuring CellTrace Violet dye dilution (**Figure 3D**). The proliferation of CD8neg and CD8+CAR T cells was not affected by oxygen conditions (**Figure 3E**), while in the corresponding CAR-negative subsets, CD8neg^Hyp-Hyp^CAR-negative cells proliferated less than ^Nor-Nor^CAR-negative cells at T1 and T2, and CD8+^Hyp-Hyp^CAR-negative cells proliferated less than ^Nor-Nor^CAR-negative cells at T2 (**Supplementary Figure 3B**). Antigen activation induced CD69 upregulation, and proliferated viable CD8neg^Hyp-Hyp^CAR T cells were more activated (CD69+) after two stimulations with cognate antigen compared to CD8neg^Nor-Nor^CAR T cells (**Figure 3F**, **Supplementary Figure 3C**).

Separately, we analyzed differentiation phenotype after stimulation with cognate antigen. At T0, CAR T cells and CAR-product exhibited similar differentiation phenotypes (**Supplementary Figure 3D**). At T1, apart from a higher CD8+ terminally differentiated (T_EMRA_) fraction in ^Nor-Nor^CAR-product, the phenotypes of ^Nor-Nor^CAR and ^Nor-Hyp^CAR-products appeared similar, predominantly comprised of effector memory cells. ^Hyp-Hyp^CAR-product was enriched in T_N_ and central memory (T_CM_) compared to ^Nor-Nor^CAR and ^Nor-Hyp^CAR-products. At T2, among both CD4+ and CD8+ cells, ^Hyp-Hyp^CAR-product was more enriched in T_N_ and less enriched in T_EM_ compared to ^Nor-Nor^CAR-product. The CD4+ ^Hyp-Hyp^CAR-product also contained more T_CM_ than ^Nor-Nor^CAR-product (**Figure 3G**).

### ^Hyp^CAR and ^Nor^CAR exhibit similar effector functions and immune checkpoint expression in response to chronic stimulation in hypoxia

**Figure 4:**
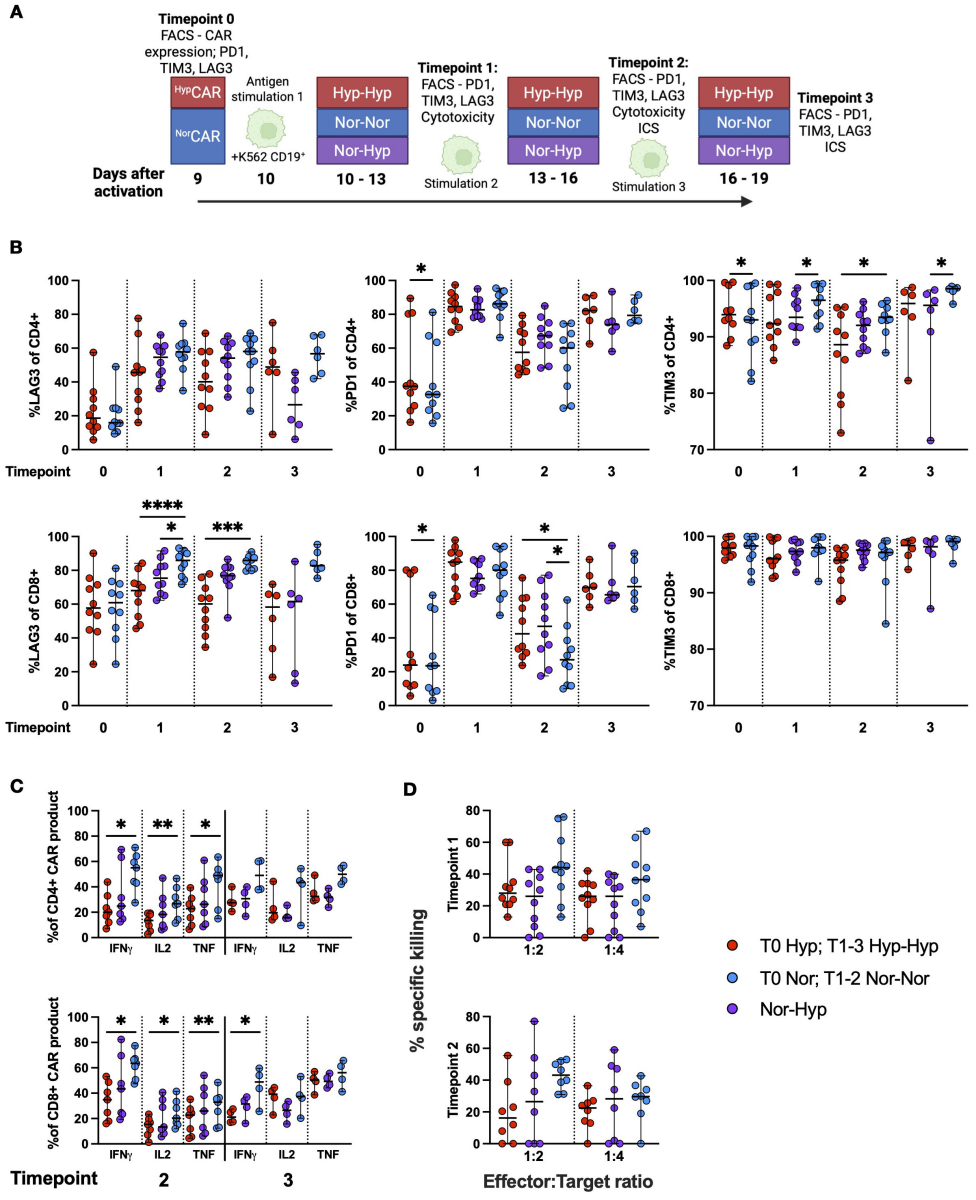
Immune checkpoint expression and function in ^Nor^CAR and ^Hyp^CAR-product after antigen challenge. **(A)** Experimental overview. **(B)** Frequency of LAG3, TIM3, and PD1 in CAR-product at T0-3. **(C)** Cytokine production by CAR-product after co-culture with K562-CD19+ at T2 and 3. **(D)** Specific killing of K562-CD19+ by CAR-product at 1:2 and 1:4 E:T ratios. Median with range are indicated. Wilcoxon tests were used for comparisons between two donor-matched conditions, and Friedman tests were used for comparisons between three donor-matched conditions. *P < 0.05, **P < 0.01, ***P < 0.001, ****P < 0.0001, n=4-10.

We set up an iterative restimulation assay (overview, **Figure 4A**) and assessed the expression (frequency) of PD1, TIM3, and LAG3 immune checkpoints after repeated stimulations with K562-CD19+ (**Figure 4B**). In the CD4+ compartment, at T0, PD1 and TIM3 were significantly increased in ^Hyp^CAR compared to ^Nor^CAR-product. At T1 and T3, TIM3 was significantly increased in CD4+^Nor-Nor^CAR as compared to ^Nor-Hyp^CAR-product; at T2, TIM3 was significantly increased in CD4+^Nor-Nor^CAR as compared to ^Hyp-Hyp^CAR-product. In the CD8+ compartment, PD1 was significantly increased in ^Hyp^CAR as compared to ^Nor^CAR-product at T0. At T1, LAG3 was significantly higher in ^Nor-Nor^CAR than in ^Nor-Hyp^CAR and ^Hyp-Hyp^CAR-products with an especially marked difference between ^Nor-Nor^CAR and ^Hyp-Hyp^CAR-product which persisted at T2. At T2, PD1 was significantly lower in ^Nor-Nor^CAR-product as compared to both ^Nor-Hyp^CAR- and ^Hyp-Hyp^CAR-products. We also evaluated the gMFI of immune checkpoints at each timepoint (**Supplementary Figure 4A**) and found, similar to the frequency assessment, no clear pattern in expression levels associated with any oxygen condition. Notably, at no point could we find any significant difference in frequency or gMFI of immune checkpoints between ^Nor-Hyp^CAR and ^Hyp-Hyp^CAR-product.

We performed intracellular cytokine staining (**Figure 4C**) and found that at T2, CD4+ and CD8+^Nor-Nor^CAR-product produced significantly more IFN-γ, IL-2, and TNF than the corresponding ^Hyp-Hyp^CAR-product when stimulated with K562-CD19+. At T3, CD8+^Nor-Nor^CAR-product produced significantly more IFN-γ than CD8+^Hyp-Hyp^CAR-product. When maximally stimulated with PMA/Ionomycin, CD4+ and CD8+^Nor-Nor^CAR-product produced significantly more IFN-γ than the corresponding ^Hyp-Hyp^CAR-product (**Supplementary Figure 4B**), and CD8+^Nor-Nor^CAR-product produced significantly more TNF than both the corresponding ^Nor-Hyp^CAR and ^Hyp-Hyp^CAR-products. At T3, CD4+^Nor-Nor^CAR-product produced significantly more IL-2 than the corresponding ^Nor-Hyp^CAR-product.

We evaluated specific killing after iterative restimulation and found no significant difference in killing capacity between oxygen conditions at either T1 or T2 at 1:2 and 1:4 E:T ratio (**Figure 4D**).

### Distinct metabolic profiles in ^Hyp^CAR and ^Nor^CAR-products after antigen-specific stimulation

**Figure 5:**
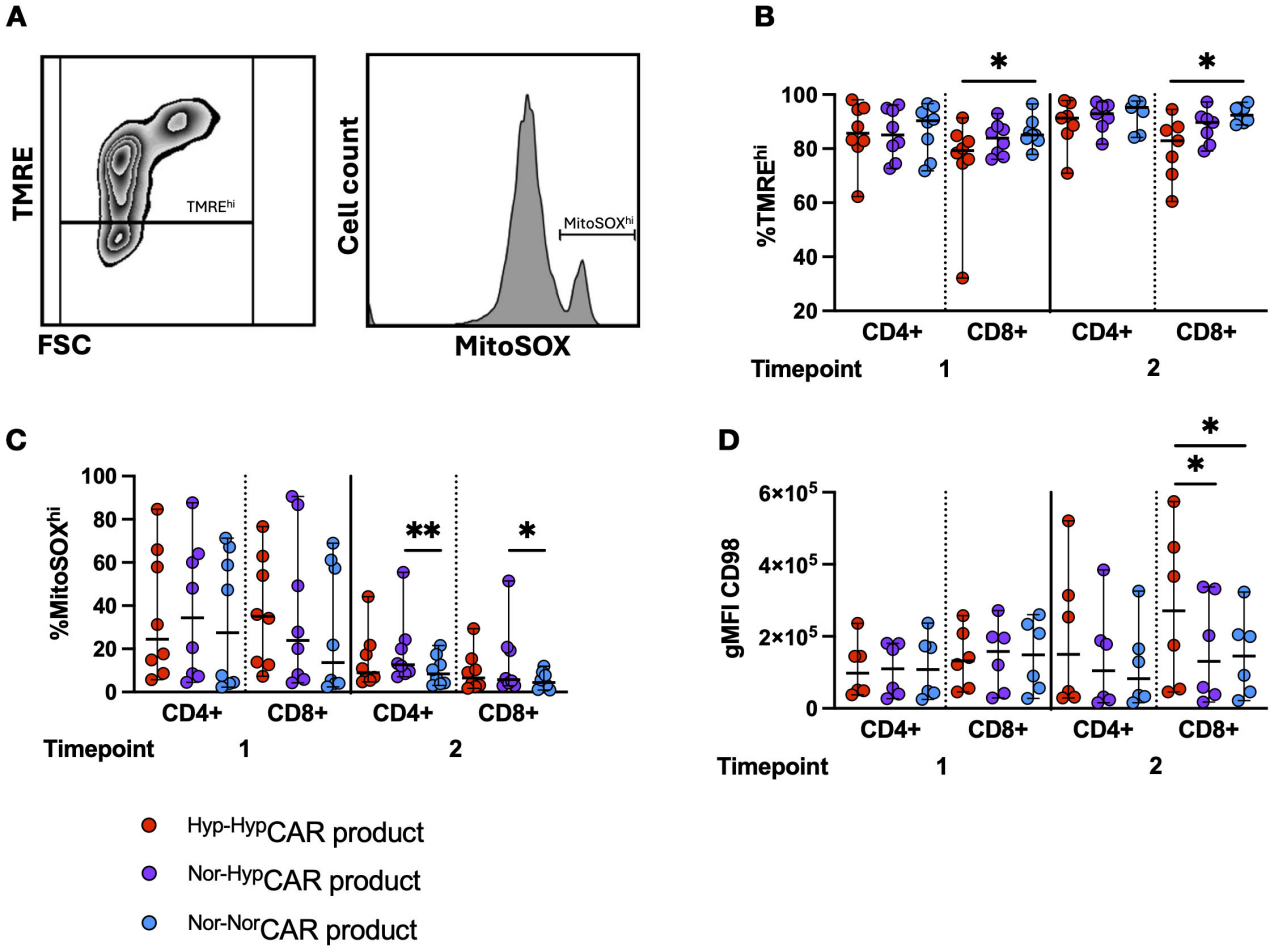
Metabolic markers in ^Nor^CAR and ^Hyp^CAR-products following antigen challenge. **(A)** Representative flow cytometry plot of polarized (TMRE^hi^) gate and MitoSOX^hi^ histogram. **(B)** Frequency of polarized cells in CAR-product at T1, T2. **(C)** MitoSOX^hi^ frequency in CAR-product at T1, T2. **(D)** CD98 gMFI in CAR-product at T1, T2. Median with range are indicated. Friedman tests used for three donor-matched conditions. *P < 0.05, **P < 0.01, n=6-8.

We used TMRE and MitoSOX dyes as proxies for mitochondrial polarization and mitochondrial reactive oxygen species (mROS) (**Figure 5A**), respectively, and assessed expression of the amino acid transporter CD98 in CAR-products stimulated with K562-CD19+ (**Figures 5B–D**) or K562-NGFR+ cells (**Supplementary Figures 5A–C**) (same experimental layout shown in **Figure 3A**). At T1 and T2, we found significantly increased mitochondrial polarization (TMRE^hi^) among ^Nor-Nor^CAR-product as compared to ^Hyp-Hyp^CAR-product. There was no difference in the frequency of MitoSOX^hi^ between oxygen states at T1, however, at T2, CD4+ and CD8+^Nor-Hyp^CAR-product were more enriched in MitoSOX^hi^ than ^Nor-Nor^CAR-product. At T1, there was no difference in the expression of CD98, but at T2, CD98 gMFI was significantly increased in CD8+^Hyp-Hyp^CAR-product compared to ^Nor-Hyp^CAR and ^Nor-Nor^CAR-products.

### Increased mitochondrial mass, potential and mROS in CLL-derived ^Hyp^CAR T cells

**Figure 6:**
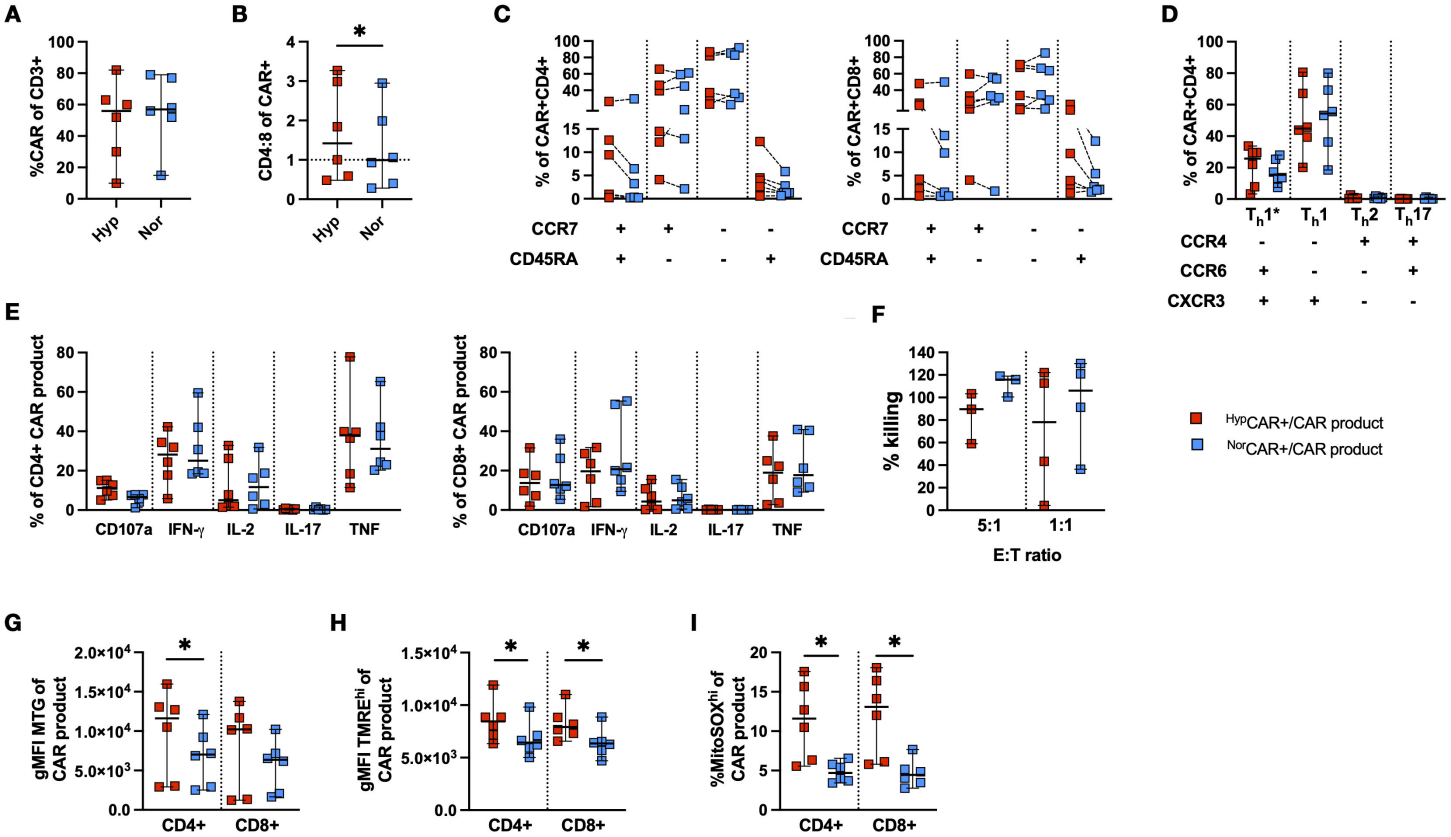
Phenotypic and functional characterization of CLL-derived CD19 CAR T cells produced in 2% and 21% O_2_. **(A)** CAR T cell frequency. **(B)** CD4:8 ratio of ^Nor^CAR and ^Hyp^CAR T cells. **(C)** Differentiation phenotype of CAR T cells. **(D)** T-helper subsets among ^Nor^CAR and ^Hyp^CAR T cells. **(E)** Cytokine production by CAR-product. **(F)** CAR-product cytotoxicity at 5:1 and 1:1 E:T ratios. **(G)** gMFI MitoTracker Green (MTG) in CAR-product. **(H)** gMFI TMRE^hi^ in CAR-product. **(I)** Frequency MitoSOX^hi^ in CAR-product. Median with 95% confidence interval are indicated Wilcoxon tests used for comparisons between two donor-matched conditions. *P < 0.05, n=3-6.

CAR T cells were produced using PBMCs from patients with CLL, in 2% and 21% O_2_ (**Figure 6A**). The CD4/CD8 ratio was higher at 2% O_2_ than at 21% (**Figure 6B**). Low frequencies of T_N_ were detected in CD4+ and CD8+CAR T cells. Oxygen conditions did not affect the differentiation phenotype (**Figure 6C**).

CAR T cells were predominantly Th1 and Th1*, and there were no differences in the proportion of CD4+ T-helper subsets between oxygen conditions (**Figure 6D**). No differences were observed in the frequency of degranulating and cytokine-producing CAR-product after stimulation with K562-CD19+ cells (**Figure 6E**). We found no difference in CAR T cell cytotoxicity between oxygen conditions at either E:T ratio (**Figure 6F**). Notably, ICS was performed in oxygen settings congruent with the production conditions, while cytotoxicity was evaluated in 21% O_2_ for all conditions. When assessing mitochondrial activity and fitness, the CD4+^Hyp^CAR-product had a higher mitochondrial mass than ^Nor^CAR-product (**Figure 6G**). Furthermore, CD4+ and CD8+^Hyp^CAR-product appeared more polarized than the corresponding ^Nor^CAR-product (**Figure 6H**). CD4+ and CD8+^Hyp^CAR-product had increased frequencies of MitoSOX^hi^ cells compared to ^Nor^CAR-product (**Figure 6I**).

## Discussion

In this study, we describe the phenotype and *in vitro* effector functions of CD19 CAR T cells produced in 2% O_2_ and generated from HDs and patients with CLL. Our results show that CD19-directed CD4+ and CD8+CAR T cells can be efficiently produced in hypoxia and that HD-derived ^Hyp^CAR T cells contain an increased frequency of naïve-like cells, along with an apparent increase in mitochondrial activity in ^Hyp^CAR T cells derived from patients with CLL.

Current knowledge suggests both positive and negative effects associated with exposure of T cells and CAR T cells to low oxygen tension. Stimulation of CD8+ T cells in hypoxia (1.5% O_2_) has been shown to drive the loss of mitochondrial function and promote a dysfunctional exhausted-like state (17), and culturing T cells in hypoxic or physiological oxygen levels (1-5% O_2_) induced decreased T cell proliferation *in vitro* (18), but also enhanced T cell anti-tumor functions *in vivo* (19). To our knowledge, only a limited number of published studies have reported the impact of restricting oxygen availability during CAR T cell production. These studies utilized CAR constructs that included CD28 (12) or 4-1BB co-stimulatory domains (13, 20) and 1% O_2_ as their benchmark for hypoxia, but the timing and duration of exposure to restricted oxygen levels varied; cells were exposed to hypoxia either during expansion (12, 20) or briefly during activation (13). In our study, ^Hyp^CAR-product was kept in a 2% O_2_ incubator for the duration of CAR T cell production, encompassing both activation and expansion phases.

As reported by others (using other CAR constructs and with either CD28 or 4-1BB co-stimulatory domains), we found that ^Hyp^CAR T cells expanded less than ^Nor^CAR T cells, CAR transduction efficiency was not affected by hypoxia, and CD4+ and CD8+^Hyp^CAR T cells displayed a less differentiated phenotype than CD4+ and CD8+^Nor^CAR T cells. A recent report (21) showed that exposure of activated T cells to 0.5% O_2_ for 48 h induced a transcriptional shift towards a resting/naïve signature, suggesting that in hypoxic conditions differentiated cells acquire a naïve-like phenotype, rather than promoting the proliferation and/or survival of naïve cells. Interestingly, we also found that naïve-like CD4+^Hyp^CAR T cells expressed significantly more CXCR3, which contributes to the regulation of T cell homing (22), than the corresponding ^Nor^CAR T cells. Hypoxia regulates Th17-regulatory T cell balance (23), but we did not observe differences between oxygen conditions in terms of the frequency of Th subsets (including Th17 and Th1*) or expression of regulatory T cell markers such as CD39 and CD73. Th1 and Th1* subsets were the most prevalent subsets in both the ^Nor^CAR and ^Hyp^CAR subsets, demonstrating that low oxygen levels did not alter the favorable Th1 polarization.

In terms of CAR T cell functionality, Berahovich et al. (12) observed decreased IFN-γ production by hypoxic BCMA and CD19 CAR T cells and increased IL-2 production by hypoxic BCMA CAR T cells. We found no differences in IL-2, IFN-γ and TNF production between ^Hyp-Hyp^CAR and ^Nor-Hyp^CAR-products. However, compared to ^Nor-Nor^CAR-product, the CD4+^Hyp-Hyp^CAR-product produced significantly more IL-2 and TNF. In a model of chronic stimulation in hypoxia, CAR T cells produced in hypoxia and normoxia show comparable effector functions. While we observed a decreased cytokine output in ^Hyp-Hyp^CAR-product at T2, but not T3, as compared to ^Nor-Nor^CAR-product, we noted comparable cytokine production between ^Hyp-Hyp^CAR- and ^Nor-Hyp^CAR-product.

*In vitro*, ^Hyp-Hyp^CAR-product had impeded cytotoxicity compared to ^Nor-Nor^CAR-product at the 1:2 E:T ratio, but not at the more stringent 1:4 to 1:10 ratios. When CAR-products were exposed to several rounds of stimulation with target cells at 1:2 and 1:4 E:T ratios, ^Hyp-Hyp^CAR-, ^Nor-Hyp^CAR- and ^Nor-Nor^CAR-products exhibited similar killing capacities. Cunha et al. (13) reported increased killing capacity in CD8+CAR T cells exposed to 1% O_2_ for one day after activation, but not in cells exposed to 1% O_2_ for three days following activation; however, the hypoxic and normoxic CAR T cells were transduced on different days (day 3 and day 1, respectively) and had subsequently been expanded for different durations (4 and 6 days in hypoxia and normoxia, respectively). Furthermore, they found that the increased *in vitro* killing capacity of CAR T cells exposed for 1 d to hypoxia also translated to increased tumor control and prolonged survival *in vivo*.

Scharping et al. have shown that expansion of activated CD8+ T cells in hypoxia and continuous stimulation resulted in an exhausted-like state (17). However, in our study, during repeated challenges with CD19+ target cells in hypoxia, ^Hyp^CAR and ^Nor^CAR-products were similar in terms of proliferative capacity, activation level, differentiation, expression of immune checkpoints, and mitochondrial activity. During the expansion of CAR T cells no stimulation is included in the cell production protocol, but CAR expression induces tonic signaling. It would thus be interesting to study the impact on T cells of simultaneous exposure to tonic signaling and hypoxia as compared to simultaneous exposure to antigen stimulation and hypoxia and decipher whether this may account for the differences (*e.g.* exhaustion state) observed between stimulated T cells and CAR T cells after expansion in hypoxia. Notably, only ^Hyp-Hyp^CAR- but not ^Nor-Hyp^CAR-product, was significantly more enriched in T_N_ cells than in ^Nor-Nor^CAR-product after several stimulations. Only CD8+^Hyp-Hyp^CAR, but not ^Nor-Hyp^CAR-product, had decreased levels of mitochondrial polarization after stimulations compared to ^Nor-Nor^CAR-product. After two stimulations with cognate antigen, CD98 expression was increased in CD8+^Hyp-Hyp^CAR-product compared to ^Nor-Nor^CAR and ^Nor-Hyp^CAR-products. CD98, an amino acid transporter that also mediates integrin-mediated signaling, is rapidly upregulated upon T cell activation. Overexpression of amino acid transporters (SLC7A5 or SLC7A11) by CAR T (24). The increased expression level of CD98 observed in CD8+^Hyp^CAR-product after repeated tumor challenges may therefore provide ^Hyp^CAR T cells with a survival advantage, particularly in the context of a nutrient-deficient TME.

T cell dysfunction is often seen in patients with CLL, since CLL tumor cells can negatively impact CD4+ and CD8+T cell mitochondrial fitness (25) and alter T cell metabolome by creating a pseudohypoxic state (26). All commercially available CAR T cell products are produced from patient-derived T cells, and strategies to improve the fitness of CAR T cells are needed, particularly in CLL. The frequency of naive-like in circulating T cells is decreased in patients with CLL as we and others have shown (14) and although the frequency of naive-like CAR T cells was very low we observed a slight increase in ^Hyp^CAR T cells. Our data demonstrate that ^Hyp^CAR T cells derived from patients with CLL display an increased frequency of CD4+ cells, comparable phenotype and, as observed with HDs, comparable effector functions in terms of cytokine production and interestingly, despite the known dysfunction of T cells in patients with CLL, high killing activity (albeit the killing assay was performed with a limited number of samples and could only be evaluated in normoxic conditions) to ^Nor^CAR T cells and, interestingly, increased mitochondrial mass, membrane potential, and mROS production. Conflicting evidence exists regarding the impact of high mitochondrial potential on T cells, high mitochondrial membrane potential and mROS have been shown to be detrimental to T cells (27), but others have shown that enhancing mitochondrial membrane potential improves CD8+ T cell function (28). In the context of CAR T cell treatment for CLL, increased mitochondrial mass in infused CD19-directed CD8+CAR T cells was shown to be higher in patients who had a complete response and was correlated with increased *in vivo* persistence (25).

There is a growing body of evidence to support that activation and/or expansion under conditions of restricted oxygen can favorably modulate T cell phenotype and effector functions (13, 29), which is consistent with our findings. We show that prolonged exposure to low oxygen levels is not necessarily detrimental to CAR T cell production, resulting in a ^Hyp^CAR-product which is enriched in Th1 and Th1* CD4+ T cells and, as compared to ^Nor^CAR-product, in naïve-like cells, a phenotype associated with superior persistence and antitumor efficacy (30). After chronic stimulation in hypoxia, ^Hyp^CAR-product retained this enrichment in naïve-like cells, and demonstrated cytokine production and cytotoxic capacity. The increased mitochondrial mass and membrane potential in CAR T cells derived from patients with CLL is of particular interest. The observation by Cunha et al. that short exposure to 1% O_2_ of *i)* CD8+ T cells increased their mitochondrial mass, membrane potential, accumulation of fatty acid metabolites and *ii)* CD8+ CAR T cells increased their *in vivo* anti-tumor function, further supports a potential increased *in vivo* efficacy of ^Hyp^CAR-T cells enriched in naïve-like cells (as observed in HDs) and/or increased mitochondrial fitness (*i.e.* mass and membrane potential, as observed in patients with CLL). Our study does not include measurement of metabolic data, analysis of the metabolic pathways utilized by CAR T cells produced from HDs and patients with CLL produced in hypoxia as compared to normoxia would provide insightful information on whether hypoxic production may indeed improve T cell functions in TMEs.

Altogether, hypoxia may represent a readily available strategy for the production of CAR T cells suited to function and persist in the hypoxic tumor microenvironment. Further investigation is however warranted, particularly *in vivo* studies comparing the anti-tumor efficacy of ^Hyp^CAR and ^Nor^CAR T cells in order to assess whether the modest impact of hypoxia that we observed in this report on CAR T cells translate into increased *in vivo* efficacy of CAR T cells in the context of pseudohypoxia induced by CLL cells.

## Data Availability

The raw data supporting the conclusions of this article will be made available by the authors, without undue reservation.

## Supplementary material

Supplementary Figure 1 Expression of surface markers associated with regulatory T cells. A. Frequency of CD25, CD39, CD73, and CD127 in CAR+ and CAR-negative T cells. Experiment performed in RPMI-1640 supplemented with 10% FBS, 1% Penicillin-Streptomycin, and 300 IU/mL IL-2. Wilcoxon tests were used for comparisons between two donor-matched conditions, n=5.

Supplementary Figure 2 Cytokine production by maximally stimulated ^Nor^CAR- and ^Hyp^CAR-product. A. Cytokine production by CAR-product after stimulation with PMA/Ionomycin. Friedman tests were used for three donor-matched conditions, n=6.

Supplementary Figure 3 CAR expression, viability, activation, and memory phenotype in ^Nor^CAR and ^Hyp^CAR-T cells in absence of antigen challenge. A. CAR expression at T0, T1, and T2 (unstimulated cells). B. Frequency of divided cells among live CAR-negative T cells at T0, T1, and T2 after stimulation with K562-CD19^+^. C. Frequency of CD69^+^ among unstimulated, divided, live CAR^+^ T cells at T0, T1, and T2. D. Differentiation phenotype of CAR^+^ T cells and CAR-product at T0. Wilcoxon tests were used for comparisons between two donor-matched conditions; Friedman tests were used for three or more. * P < 0.05, ** P < 0.01 n=6.

Supplementary Figure 4 Immune checkpoint expression and cytokine production after chronic stimulation. A. gMFI of LAG3, PD1, and TIM3 in CAR-product at T0-3. B. Cytokine production in response to stimulation with PMA/Ionomycin. Wilcoxon tests were used for comparisons between two donor-matched conditions; Friedman tests were used for three donor-matched conditions. * P < 0.05, ** P < 0.01, *** P < 0.001, n=4-10.

Supplementary Figure 5 Metabolic markers in ^Nor^CAR- and ^Hyp^CAR-products following non-specific antigen challenge. A. %TMRE^hi^ in non-specifically stimulated CAR-product at T1 and T2. B. %MitoSOX^hi^ in non-specifically stimulated CAR-product at T1 and T2. C. gMFI of CD98 in CAR-product at T1 and T2. Friedman tests were used for three donor-matched conditions. * P < 0.05, n=5-8.
