# Supplementary figures and images for: Production of functional CD19 CAR T cells under hypoxic manufacturing conditions
Source: Front Immunol. 2025 Oct 8;16:1675786. doi: 10.3389/fimmu.2025.1675786 (PMC12540424; doi:10.3389/fimmu.2025.1675786)

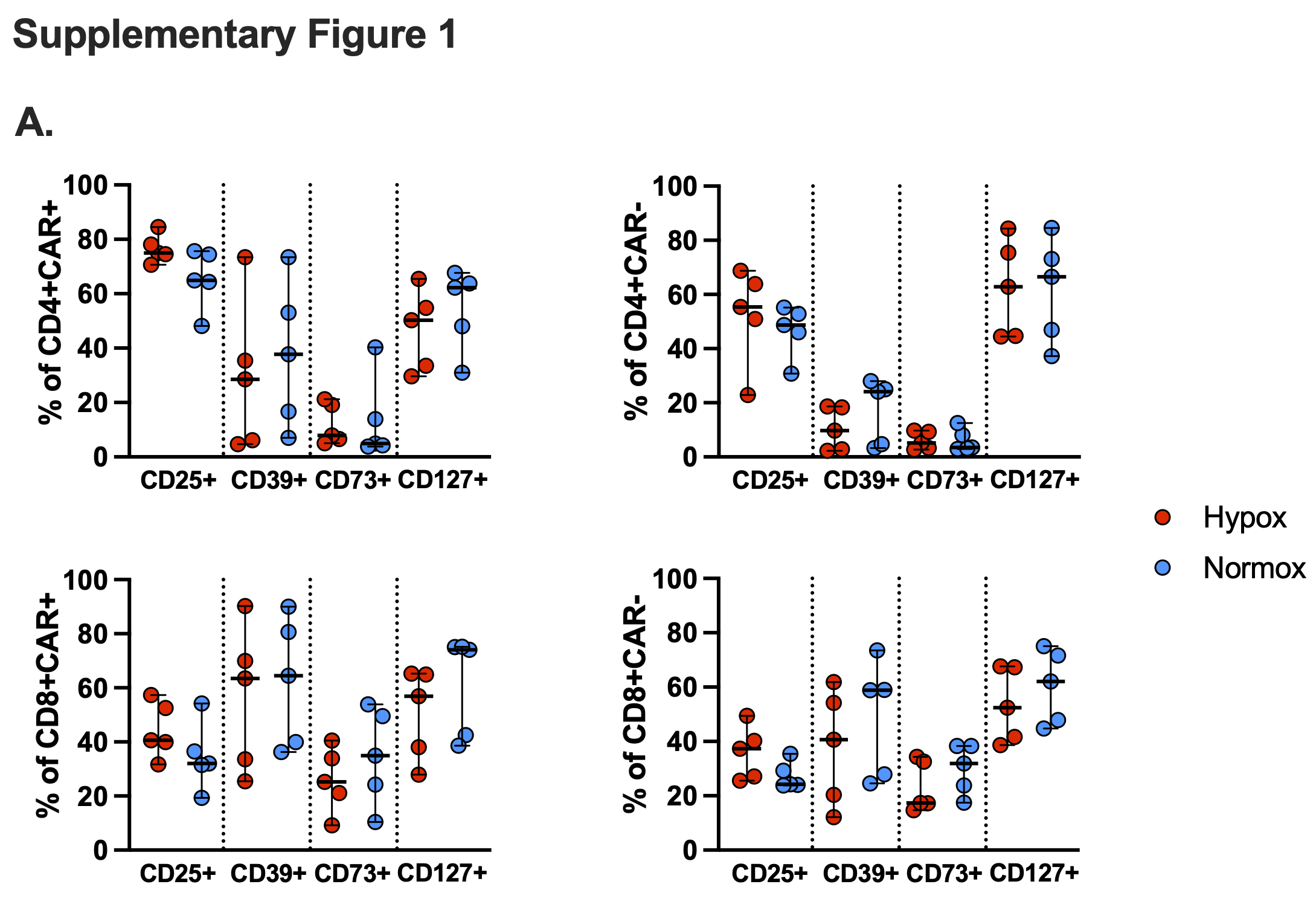

Supplement: Supplementary Figure 1 — Expression of surface markers associated with regulatory T cells. A. Frequency of CD25, CD39, CD73, and CD127 in CAR+ and CAR-negative T cells. Experiment performed in RPMI-1640 supplemented with 10% FBS, 1% Penicillin-Streptomycin, and 300 IU/mL IL-2. Wilcoxon tests were used for comparisons between two donor-matched conditions, n=5. [file Image1.tiff]

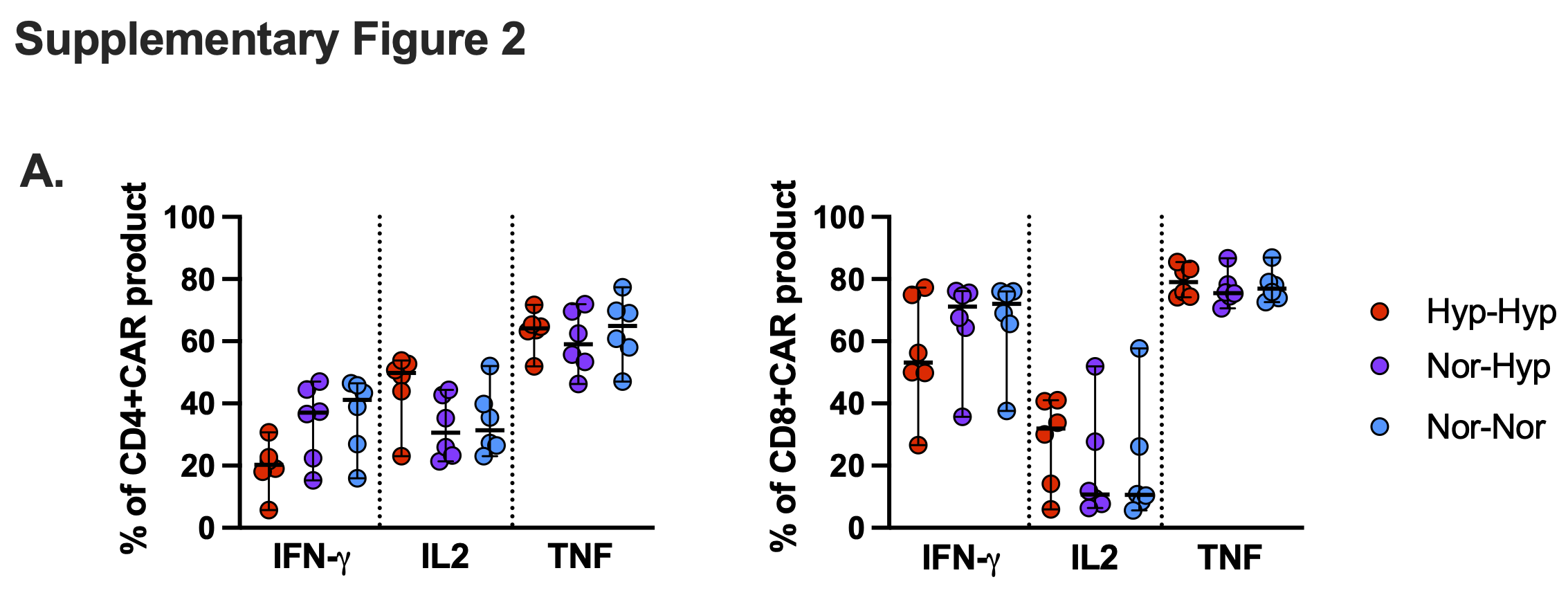

Supplement: Supplementary Figure 2 — Cytokine production by maximally stimulated NorCAR- and HypCAR-product. A. Cytokine production by CAR-product after stimulation with PMA/Ionomycin. Friedman tests were used for three donor-matched conditions, n=6. [file Image2.tiff]

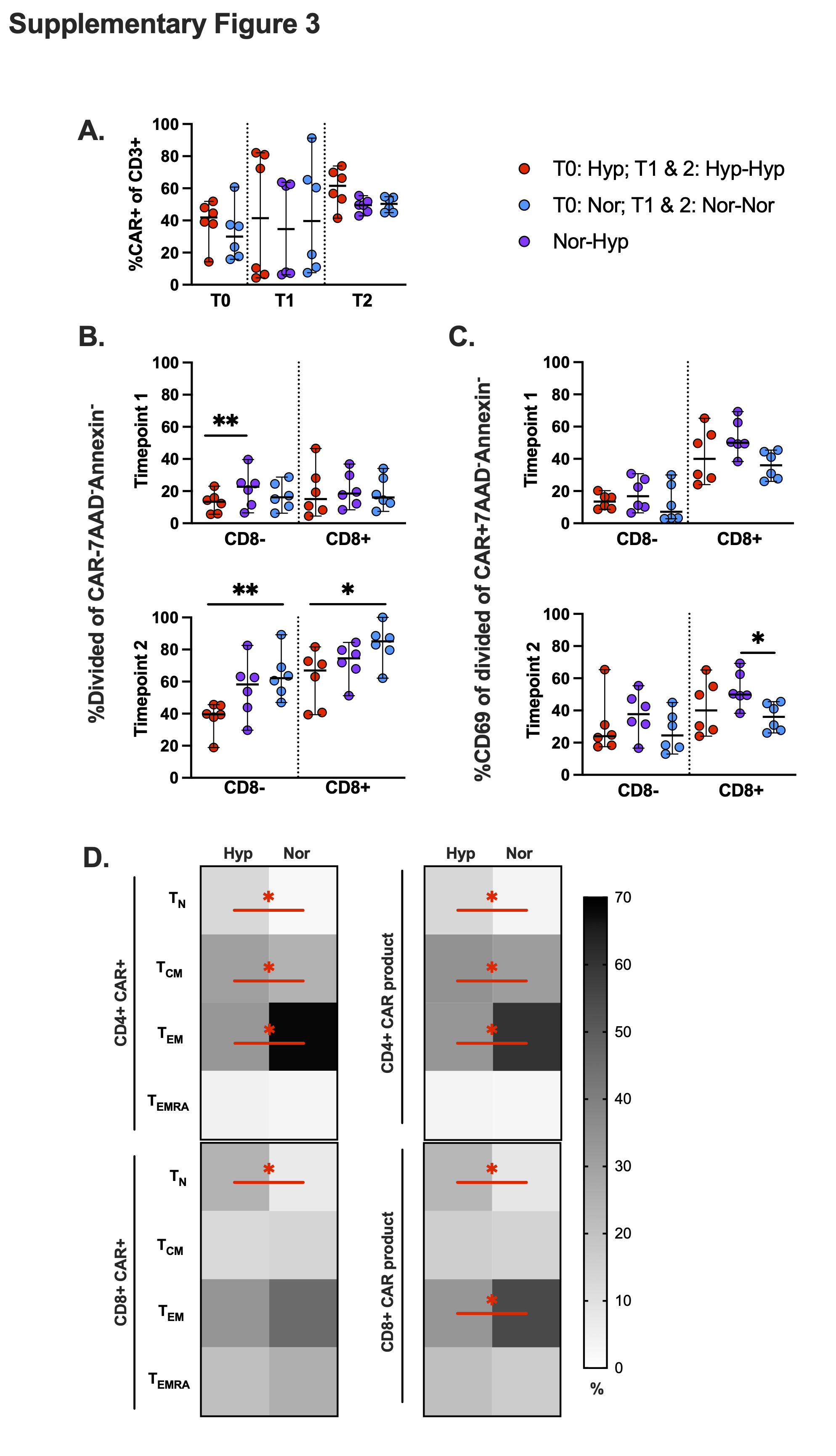

Supplement: Supplementary Figure 3 — CAR expression, viability, activation, and memory phenotype in NorCAR and HypCAR-T cells in absence of antigen challenge. A. CAR expression at T0, T1, and T2 (unstimulated cells). B. Frequency of divided cells among live CAR-negative T cells at T0, T1, and T2 after stimulation with K562-CD19+. C. Frequency of CD69+ among unstimulated, divided, live CAR+ T cells at T0, T1, and T2. D. Differentiation phenotype of CAR+ T cells and CAR-product at T0. Wilcoxon tests were used for comparisons between two donor-matched conditions; Friedman tests were used for three or more. * P < 0.05, ** P < 0.01 n=6. [file Image3.tiff]

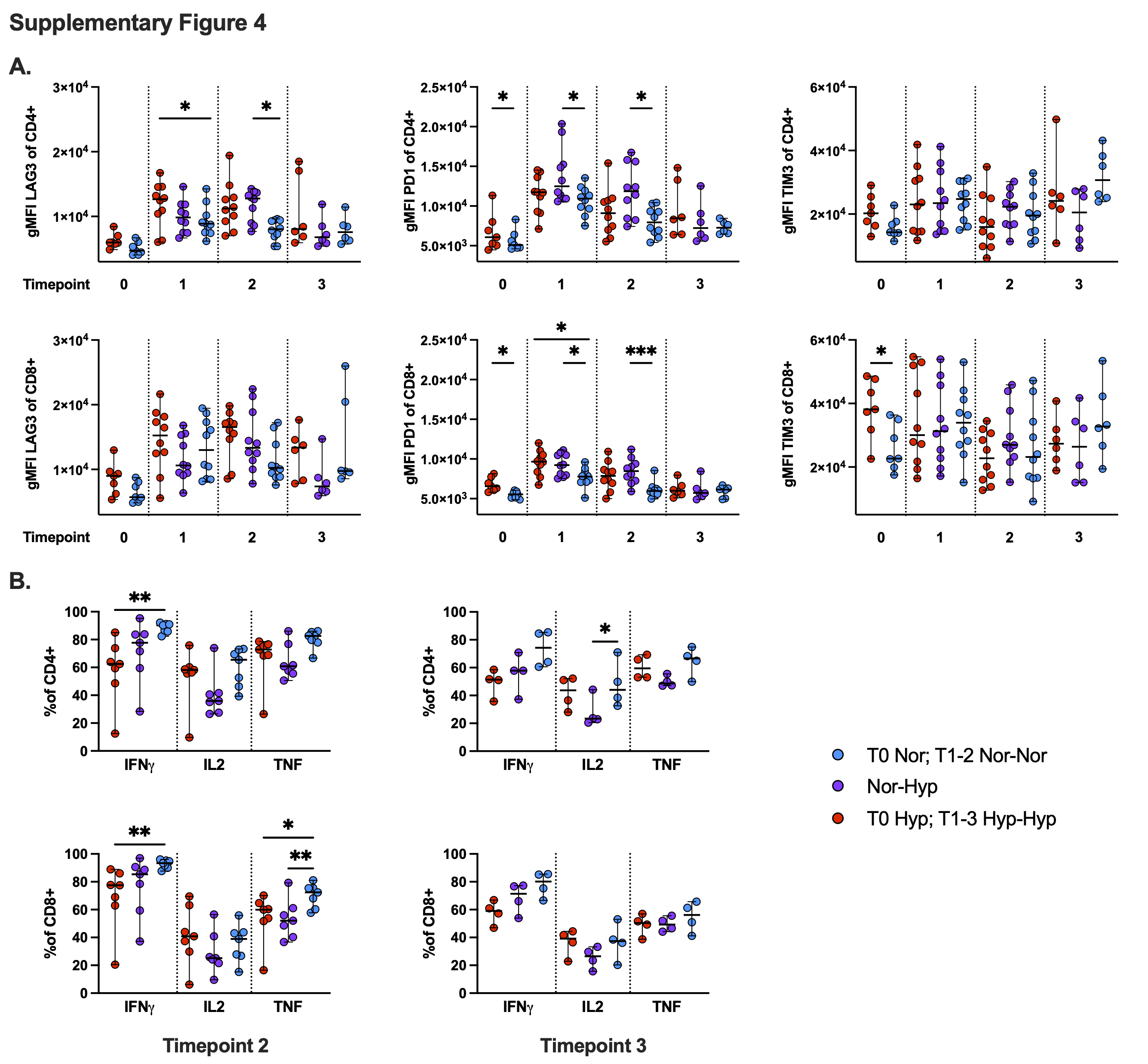

Supplement: Supplementary Figure 4 — Immune checkpoint expression and cytokine production after chronic stimulation. A. gMFI of LAG3, PD1, and TIM3 in CAR-product at T0-3. B. Cytokine production in response to stimulation with PMA/Ionomycin. Wilcoxon tests were used for comparisons between two donor-matched conditions; Friedman tests were used for three donor-matched conditions. * P < 0.05, ** P < 0.01, *** P < 0.001, n=4-10. [file Image4.tiff]

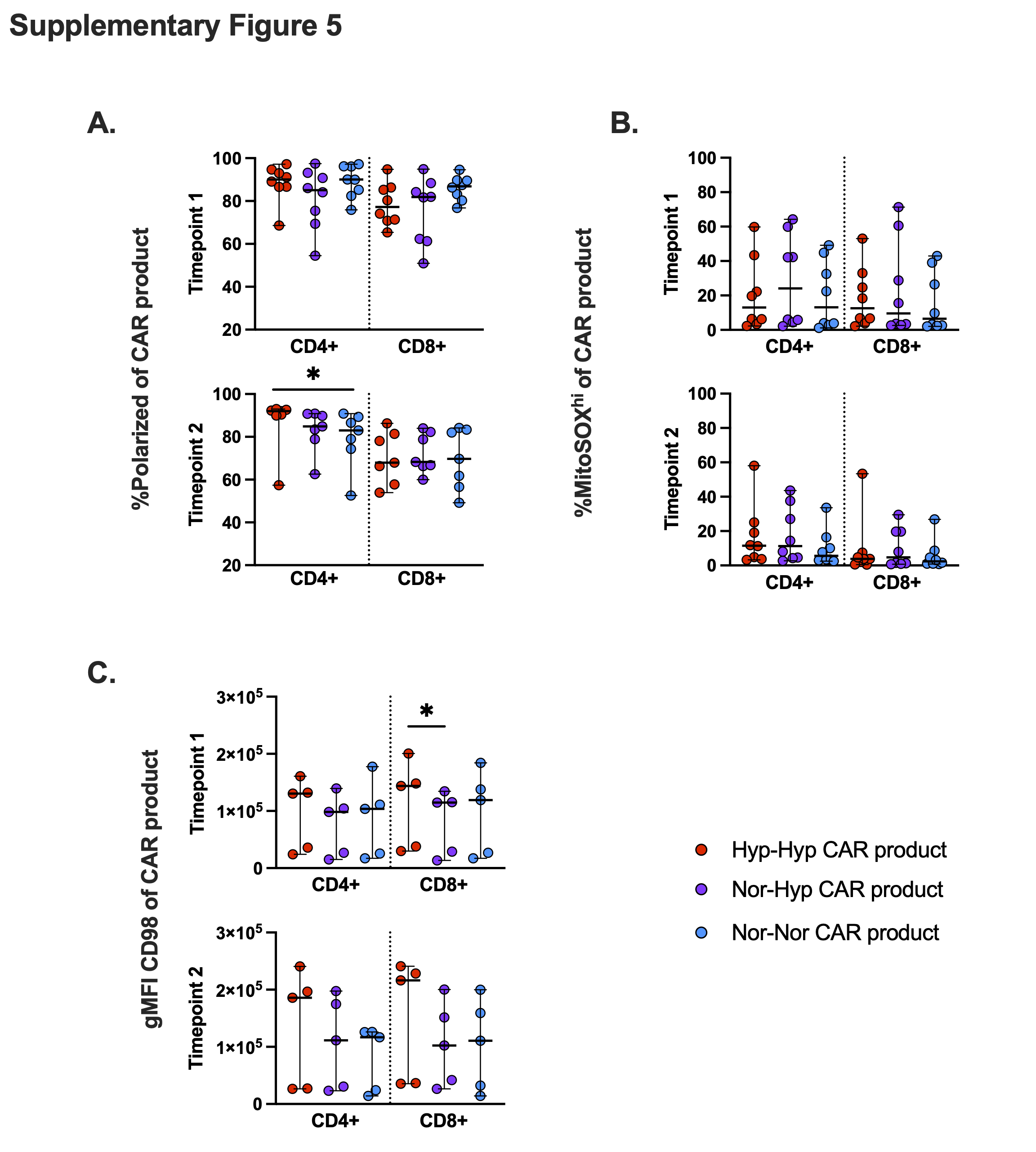

Supplement: Supplementary Figure 5 — Metabolic markers in NorCAR- and HypCAR-products following non-specific antigen challenge. A. %TMREhi in non-specifically stimulated CAR-product at T1 and T2. B. %MitoSOXhi in non-specifically stimulated CAR-product at T1 and T2. C. gMFI of CD98 in CAR-product at T1 and T2. Friedman tests were used for three donor-matched conditions. * P < 0.05, n=5-8. [file Image5.tiff]
