# Supplementary material for: Production of functional CD19 CAR T cells under hypoxic manufacturing conditions
Source: Front Immunol. 2025 Oct 8;16:1675786. doi: 10.3389/fimmu.2025.1675786 (PMC12540424; doi:10.3389/fimmu.2025.1675786)
Supplement: Supplementary file 6 [file Table1.docx]

**Supplementary material**

Supplementary Table 1. CD19 CAR expression by flow cytometry

| Specificity | Conjugation | Vendor |
| --- | --- | --- |
| Goat anti-mouse IgG (anti-CAR) | Biotin | Jackson Immunoresearch |
| Streptavidin | PE | Biolegend |
