# Supplementary material for: Production of functional CD19 CAR T cells under hypoxic manufacturing conditions
Source: Front Immunol. 2025 Oct 8;16:1675786. doi: 10.3389/fimmu.2025.1675786 (PMC12540424; doi:10.3389/fimmu.2025.1675786)
Supplement: Supplementary file 7 [file Table2.docx]

**Supplementary material**

Supplementary Table 2. Flow cytometry antibodies

| Specificity | Conjugation | Vendor |
| --- | --- | --- |
|  |  |  |
| Annexin V | FITC | BD Pharmingen |
| CCR4 | AF647 | BD Pharmingen |
| CCR6 | PE-CF594 | BD Pharmingen |
| CCR7 | BV421 | Biolegend |
| CD3 | ECD | Beckman Coulter |
| CD3 | PE-Cy7 | BD Pharmingen |
| CD4 | AF700 | BD Pharmingen |
| CD4 | BV510 | Biolegend |
| CD4 | FITC | BD Pharmingen |
| CD8 | APC-Cy7 | BD Pharmingen |
| CD25 | APC | BD Pharmingen |
| CD39 | BV421 | BD Pharmingen |
| CD45-RA | BV785 | Biolegend |
| CD45-RA | PercP Cy5.5 | Biolegend |
| CD69 | APC | Biolegend |
| CD73 | Per-CP eFluor | BD Pharmingen |
| CD98 | PeVio770 | Miltenyi Biotec |
| CD107a | BV510 | BD Pharmingen |
| CD127 | APC-AF700 | BD Pharmingen |
| CXCR3 | FITC | BD Pharmingen |
| IFN-γ | APC | BD Pharmingen |
| IL-2 | AF700 | Biolegend |
| IL-17 | PerCP-Cy5.5 | BD Pharmingen |
| LAG3 | BV650 | Biolegend |
| PD1 | FITC | BD Pharmingen |
| TIM3 | APC | Miltenyi Biotec |
| TNF | eFluor 610 | Invitrogen |
