# Supplementary material for: Production of functional CD19 CAR T cells under hypoxic manufacturing conditions
Source: Front Immunol. 2025 Oct 8;16:1675786. doi: 10.3389/fimmu.2025.1675786 (PMC12540424; doi:10.3389/fimmu.2025.1675786)
Supplement: Supplementary file 8 [file Table3.docx]

**Supplementary material**

Supplementary Table 3. Flow cytometry dyes

| Dye | Marker | Vendor |
| --- | --- | --- |
| CellTrace Violet | Proliferation | Invitrogen |
| 7AAD | Viability | BD Pharmingen |
| MitoSOX | mROS | Invitrogen |
| MitoTracker Green | Mitochondrial mass | Invitrogen |
| TMRE | Mitochondrial polarization | Invitrogen |
